# Evolutionary and functional diversification of the *DOG* gene family in wheat (*Triticum aestivum* L.) and their roles in seed dormancy

**DOI:** 10.1186/s12864-025-12071-1

**Published:** 2025-09-29

**Authors:** Yongjing Ni, Can Guo, Xiuyin Zhang, Mengjin Ma, Xuemin Liu, Yanping Li, Shaoying Lian, Junchang Li, Yumei Jiang, Jishan Niu, Lei Li

**Affiliations:** 1 Shangqiu Academy of Agricultural and Forestry Sciences, Shangqiu, Henan 476000 China; 2 The Suihuang Laboratory, Shangqiu, Henan 476000 China; 3 Henan Province Key Laboratory of Efficient Crop Production and Food Quality Safety, Zhoukou Normal University, Zhoukou, Henan 466001 China; 4 National Engineering Research Center for Wheat/Henan Technology Innovation Centre of Wheat, Henan Agricultural University, Zhengzhou, Henan 450046 China

**Keywords:** *Triticum aestivum* L., *DOG* gene family, Expression pattern, Abiotic stress, Pre-harvest sprouting

## Abstract

**Background:**

Seed germination is a critical developmental process in plants, regulated by numerous genetic factors. The *delay of germination 1* (*DOG*) gene family plays a pivotal role in controlling seed dormancy and germination in various species. However, as a heterohexaploid (AABBDD), the genome structure of wheat (*Triticum aestivum* L.) is complex, and the evolutionary process, functional differentiation and association mechanism of the *DOG* gene family with pre-harvest sprouting (PHS) have not been clarified, which urgently needs to be systematically analyzed.

**Results:**

In this study, we conducted a comprehensive genome-wide analysis of the *DOG* gene family in wheat, identifying 53 *DOG* family members (designated *TaDOG1* ~ *TaDOG53*). Phylogenetic analysis grouped wheat, Arabidopsis, rice, and maize *DOG* proteins into three clades, with wheat genes showing closer relationships to monocots (rice/maize) than to Arabidopsis. Structural diversity, including motif composition and exon-intron organization, suggested functional specialization: Group Ⅰ genes (8 ~ 12 exons) harbored more regulatory motifs, while Group Ⅲ (1 ~ 2 exons) had minimal structures. Promoter analysis revealed abundant ABA (22.52%) and MeJA responsive (36.56%) *cis*-elements, consistent with hormone-treated expression dynamics: Synteny analysis indicated segmental duplications drove wheat *DOG* expansion, with 124 intra-genomic events and strong synteny with rice/maize. RT-PCR showed distinct temporal patterns under ABA/MeJA, with early (1 ~ 4 h) and delayed (12 h) responders mediating phased signaling. RNA-seq shows *TaDOG* genes have diverse expressions in 8 wheat tissues, clustered into 4 groups with distinct expression and roles, with grain-specific genes (*TaDOG3*) highlighting potential roles in seed dormancy. It was found that there is a negative correlation between *TaDOG3* expression and PHS rate, and *TaDOG3* can be used as a marker gene to detect the degree of spike germination.

**Conclusion:**

This study systematically analyzed the evolutionary history and functional differentiation of the wheat *TaDOG* gene family, confirming that the *TaDOG* genes play a multi-level role in seed dormancy regulation through differentiated hormone response patterns and tissue expression characteristics. Among them, *TaDOG3*, as a potential molecular marker of PHS resistance, provides a theoretical basis and practical target for wheat anti-spike germination breeding.

**Supplementary Information:**

The online version contains supplementary material available at 10.1186/s12864-025-12071-1.

## Background

Seed germination is a complex biological process that marks the transition from dormancy to active growth, crucial for plant survival and agricultural productivity [1]. It is tightly regulated by environmental cues (e.g., temperature, moisture, light) and endogenous signals, with hormonal pathways-particularly abscisic acid (ABA) and gibberellins (GA) - playing central roles [2, 3]. Genetic studies in model plants like Arabidopsis thaliana have identified numerous key regulators of seed dormancy and germination, including transcription factors, hormone signaling components, and structural proteins [4]. Among these, the Delay of germination 1 (*DOG1*) gene was first identified in Arabidopsis as a major quantitative trait locus (QTL) controlling seed dormancy [5], with mutations in *DOG1* leading to reduced dormancy and premature germination [6]. *DOG1* is closely associated with the degree of dormancy in freshly harvested seeds [7].

The discovery and functional research of *DOG1* are regarded as significant breakthroughs in seed dormancy studies [5]. There are five *DOG1*-like genes (*DOGL1 ~ 5*) in the Arabidopsis genome [8]. *DOGL1 ~ 4* are located on chromosome 4 and adjacent to each other, while *DOGL5* is located on chromosome 3. The amino acid sequences of DOGL1, DOGL2 and DOGL3 are relatively similar to those of DOG1, while the amino acid sequences of DOGL4 and DOGL5 are only 28% and 30% similar to those of DOG1, respectively [8]. Among the studied dicotyledonous plants, the amino acid sequence similarity between DOG1 is relatively high [9]. Up to now, *DOG1* genes have been identified in various plant species, including *Triticum aestivum* L., *Oryza sativa* L., *Zea mays* L., *Hordeum vulgare* L., and *Sorghum bicolor* (L.) Moench and *Brachypodium distachyon* (L.) P. Beauv [10]. In general, the higher the expression level of the *DOG1* gene, the deeper the degree of seed dormancy, and it is an important regulatory factor for seed dormancy [11]. *OsDOG1L-3* is the homologous gene of Arabidopsis *DOG1*; overexpression or introduction of Os*DOG1*L-3 allele in rice enhanced seed dormancy, *OsDOG1L-3* expression was positively correlated with seed dormancy [12]. *TaDOG1L1* can induce seed dormancy in wheat, yet it shares low sequence similarity with Arabidopsis *DOG1*, specific expression pattern of *DOG1* is not conservative in different plants [13]. In Arabidopsis, ectopic overexpression of *TaDOG1L1* and *TaDOG1L4*, respectively, significantly increased seed dormancy, and the dormancy release during dry seed storage was similar to that of transgenic plants overexpressing *DOG1* [13]. It indicates that these *DOG1*-like proteins have conserved functions in enhancing seed dormancy. Besides, Overexpression of *TaDOG1L4* in wheat. Resulted in a deeper degree of seed dormancy, while silencing of *TaDOG1L4* by RNAi resulted in a decrease in seed dormancy [14].

Seed dormancy is intricately regulated by phytohormonal networks and genetic determinants. To date, nine major phytohormones have been identified and characterized in this regulatory context, including abscisic acid (ABA), auxin (IAA), brassinosteroids (BR), cytokinins (CK), ethylene (ET), gibberellins (GA), methyl jasmonate (MeJA), salicylic acid (SA), and strigolactones (SL) [15]. Among them, ABA and MeJA represent the two most critical phytohormones governing seed dormancy, with the dynamic equilibrium between ABA and MeJA being central to the regulatory network controlling seed dormancy maintenance and germination initiation [2]. Despite a few reports showing the role of MeJA in regulating seed dormancy, JA inhibits seed germination synergistically with ABA [16]. The *DOG1* gene, as a negative regulatory factor, regulates seed dormancy by altering endogenous hormone balance through the ABA pathway [17]. Existing research indicates that exogenous application of ABA promotes the expression of the *DOG1* gene, increases the sensitivity of seeds to ABA, and thereby promotes seed dormancy [18].

Wheat (*T. aestivum*) is a hexaploid (AABBDD) cereal crop with a large and complex genome (~ 17 Gb), making genome-wide gene family analyses challenging but essential for understanding its agronomic traits [19]. At present, several QTLs associated with seed dormancy and pre-harvest sprouting (PHS) have been mapped in wheat [20], while the molecular mechanisms underlying these traits remain poorly understood, especially regarding the wheat *DOG* gene family. The *DOG1* gene is highly correlated with seed dormancy. However, the characteristics, and expression patterns of the *DOG* gene family in wheat, as well as the molecular mechanisms by which it responds to exogenous hormones and regulates seed dormancy, are not yet fully understood.

In this study, wheat *DOG* gene family members were identified in the Chinese Spring genome, followed by comprehensive analyses of their evolutionary relationships, structural characteristics, and *cis*-acting regulatory elements. Genome-wide collinearity analysis was performed to characterize collinearity patterns within wheat and cross-species collinearity with other organisms. Expression profiling was conducted to examine their tissue-specific expression patterns and transcriptional responses to MeJA/ABA treatments. These analyses provide novel insights into the potential roles of the *DOG* genes in regulating wheat development and stress adaptive responses.

## Materials and methods

### Plant materials and growth conditions

Wheat (*T. aestivum* cv. Chinese Spring) seeds were germinated on moist filter paper at 22 °C in the dark. Seedlings were grown in a growth chamber with a 16 h light/8 h dark cycle, 60% humidity, and 23 °C temperature. Tissues (root, stem, leaf, spike, and developing grains at the anthesis stage) were harvested, frozen in liquid nitrogen, and stored at −80 °C for RNA extraction [21].

### Genome-wide identification of the DOG genes in wheat

The wheat reference genome (IWGSC RefSeq v1.1) and protein sequences were downloaded from the *EnsemblPlants* database (http://www.plants.ensembl.org/Triticum_aestivum/Info/Index). The Arabidopsis *DOG1* protein sequence (AT5G45830) was used as a query to perform BLASTP searches (E-value < 1e-10) against the wheat proteome [22]. Additionally, Hidden Markov model (HMM) searches were conducted using the conserved domain of *DOG1* (obtained from Pfam database, PF14144) with HMMER 3.0 software [23]. Redundant sequences were removed by clustering at 95% identity, and candidate genes were verified for the presence of conserved domains using CDD (https://www.ncbi.nlm.nih.gov/Structure/bwrpsb/bwrpsb.cgi) and SMART (http://www.smart.embl-heidelberg.de/).

### Phylogenetic and sequence analysis

Full-length protein sequences of wheat *DOG* genes, along with *DOG* homologs from *Arabidopsis*, rice, and maize, were aligned using MEGA v7 (http://www.megasoftware.net/). A phylogenetic tree was constructed using Maximum Likelihood (ML) method, with 1000 bootstrap replicates, and poisson model. Conserved motifs were identified using MEME online tool (http://memesuite.org/tools/meme), with parameters set to discover 10 motifs, ranging in length from 6 to 50 amino acids. Gene structure analysis was performed by aligning DNA sequences using TBtools software [24].

### *Cis*-acting element prediction

Promoter regions (2 kb upstream of the transcription start site) of wheat *DOG* genes were extracted from the genomic sequence, and *cis*-acting elements were predicted using PlantCARE (http://www.bioinformatics.psb.ugent.be/webtools/plantcare/html). Elements related to hormone response (ABA, MeJA, auxin, etc.), stress response (drought, cold, salt), and developmental regulation were annotated and visualized.

### Genome collinearity analysis

All wheat *DOG* genes were mapped to wheat chromosomes using Circos [25]. Intra-genomic collinearity of wheat *DOG* genes was analyzed using MCScanX software, with default parameters [26]. Collinear gene pairs were identified, and duplication types (segmental, tandem, or dispersed) were classified. Synteny analysis between wheat and Arabidopsis/rice/maize was performed by BLASTP (E-value < 1e-5) and visualized using the Multiple Systeny Plot software (https://www.github.com/CJ-Chen/TBtools).

### Expression pattern analysis

RNA-seq data from various wheat tissues (root, stem, leaf, spike, and developing grain) were obtained from the Wheat Expression Browser (http://www.wheat-expression.com/). The gene expression level was quantified by the transcripts per million (TPM) values. The seedling at three-leaf stage (the 45th day after wheat sowing), root, stem, leaf, stamen, pistil, spike, and grain at the anthesis stage (the 215th day after wheat sowing) of Chinese Spring were used to carry out RT-PCR. A detailed description of Chinese Spring is provided in Section: Plant materials and growth conditions.

For hormone treatment experiments, 7-day-old seedlings were sprayed with 100 µM MeJA or 100 µM ABA, and leaf samples were collected at 0, 3, 6, 12, and 24 h. Total RNA was extracted using TRIzol reagent, reverse-transcribed into cDNA, and RT-PCR was performed with SYBR Green Master Mix on a Real-Time System (Bio-Rad). The wheat *Actin* gene was used as an internal control, and relative expression was calculated using the 2^−ΔΔCT^ method [27]. All primers were designed using software Primer Premier 5.0 (http://www.premierbiosoft.com/primerdesign/index.html) and the primer sequences are listed in Table S1. Other detailed description was carried out as described in the previous study [28].

### Pre-harvest sprouting test and verification

A total of four Chinese winter wheat varieties were selected and planted at the Shuangba Town Experimental Base (115°71′ E, 34°53′ N) of Shangqiu Academy of Agricultural and Forestry Sciences in the 2022–2023. There are Shangnong 5 (unreleased), Shangnong 6 (released no. Yushenmai 20220077), Shangnong 8 (released no. Yushenmai 20220080), and Shangnong 9 (released no. Guoshenmai 20243004) with a highly similar genetic background. A randomized block design was used with 3 biological replicates, 30 plants were planted in each plot and uniformly managed in the field to ensure minimal interference of environmental factors on the phenotype [29].

In the early stage of wheat maturity (7 to 10 days before harvest), 10 plump and disease-free wheat spikes are selected from each of the three repeated plots of each variety to conduct the PHS test. After removing impurities from the surface of the glume, they are placed in petri dishes lined with moist filter paper and kept moist in a constant temperature incubator at 25℃ for 5 days (distilled water is supplemented daily to keep the filter paper moist). After the cultivation was completed, the germination phenotype of the grains was photographed, and the morphological differences such as bud length and density of the germinated grains were recorded.

At the late grain filling stage (25 days after anaphase), kernels (30 kernels per replicate, and mixed into one biological replicate) from the middle of the panicle from three replicate plots of each variety were immediately put into liquid nitrogen snap frozen for RNA extraction, then used to detect the changes in the expression levels of marker genes related to PHS. A detailed description of RT-PCR is provided in Section: Expression pattern analysis.

### Statistical analysis

All experimental data were subjected to statistical analysis, with results presented as the mean ± standard error (SE). Each reaction in RT-PCR was performed with three biological replicates. For the seed germination rate assessment in the PHS trial, there were 10 replicates per sample. Statistical significance was evaluated using Student’s *t*-tests, different letters indicate a significant difference between samples (*P* < 0.05).

## Results

### Identification of the DOG gene family members in wheat

A total of 53 *DOG* gene family members were identified in the wheat genome, designated as *TaDOG1* to *TaDOG53* (Table 1). Sequence analysis revealed that *TaDOG* proteins ranged in length from 198 to 580 amino acids, with molecular mass of 21.66 to 62.92 kDa and isoelectric points (pI) from 5.24 to 10.37. All members contained the conserved *DOG1* domain (PF14144). Chromosomal mapping showed that these genes were unevenly distributed across all 21 wheat chromosomes, with chromosome 3 containing the most (15 genes) (Table 1).

**Table 1:** The information of the wheat *DOG* gene family

Gene name	Gene ID	Chromosome	Physical position	Protein prediction		
				Amino acid (aa)	Molecular mass (kDa)	Isoelectric point
*TaDOG1*	TraesCS1A02G096300.1	1 A	91,602,861–91,604,969	335	36.68	5.71
*TaDOG2*	TraesCS1A02G276600.2	1 A	471,695,008–471,701,095	526	58.10	6.22
*TaDOG3*	TraesCS1A02G392200.1	1 A	558,704,272–558,705,248	226	24.45	5.47
*TaDOG4*	TraesCS1B02G127400.1	1B	156,565,523–156,567,675	340	37.26	6.14
*TaDOG5*	TraesCS1B02G285800.1	1B	497,124,690–497,131,575	526	58.07	5.79
*TaDOG6*	TraesCS1B02G420300.1	1B	643,278,391–643,279,367	226	24.45	5.65
*TaDOG7*	TraesCS1D02G105300.1	1D	97,214,357–97,217,038	339	37.11	6.56
*TaDOG8*	TraesCS1D02G276100.2	1D	372,147,878–372,155,789	526	58.13	5.91
*TaDOG9*	TraesCS1D02G400300.1	1D	466,493,735–466,494,723	226	24.49	5.65
*TaDOG10*	TraesCS2A02G010800.1	2 A	4,113,715–4,114,311	198	21.66	9.56
*TaDOG11*	TraesCS2A02G097500.2	2 A	51,493,493–51,500,017	336	37.46	9.09
*TaDOG12*	TraesCS2A02G486100.1	2 A	721,468,836–721,469,585	249	27.38	10.37
*TaDOG13*	TraesCS2A02G526300.1	2 A	746,682,082–746,688,211	419	46.27	6.24
*TaDOG14*	TraesCS2B02G113200.2	2B	76,816,411–76,823,435	336	37.46	9.09
*TaDOG15*	TraesCS2B02G512700.1	2B	708,124,548–708,125,270	240	26.18	9.2
*TaDOG16*	TraesCS2B02G556600.1	2B	751,317,909–751,324,133	435	48.38	6.23
*TaDOG17*	TraesCS2D02G096800.2	2D	49,195,871–49,202,805	336	37.47	9.09
*TaDOG18*	TraesCS2D02G529000.1	2D	616,525,223–616,530,794	425	46.84	5.86
*TaDOG19*	TraesCS3A02G103500.1	3 A	67,123,770–67,124,901	260	27.87	5.6
*TaDOG20*	TraesCS3A02G190700.2	3 A	238,676,623–238,691,290	332	36.92	7.79
*TaDOG21*	TraesCS3A02G306200.1	3 A	542,734,930–542,736,059	296	31.82	5.63
*TaDOG22*	TraesCS3A02G334100.1	3 A	579,377,243–579,383,014	475	51.77	6.53
*TaDOG23*	TraesCS3A02G372400.2	3 A	623,047,081–623,054,386	573	62.14	6.64
*TaDOG24*	TraesCS3B02G120900.1	3B	91,147,416–91,148,571	260	27.94	5.43
*TaDOG25*	TraesCS3B02G220400.2	3B	269,247,185–269,257,631	332	36.92	7.79
*TaDOG26*	TraesCS3B02G330400.1	3B	534,515,809–534,517,125	295	31.80	5.24
*TaDOG27*	TraesCS3B02G365100.1	3B	576,449,738–576,455,465	477	51.92	6.78
*TaDOG28*	TraesCS3B02G404800.2	3B	640,060,963–640,068,645	573	62.06	6.45
*TaDOG29*	TraesCS3D02G105800.1	3D	58,107,658–58,108,963	260	27.98	5.46
*TaDOG30*	TraesCS3D02G194800.2	3D	187,831,578–187,841,857	332	36.93	8.49
*TaDOG31*	TraesCS3D02G295800.1	3D	408,655,818–408,656,967	292	31.49	5.37
*TaDOG32*	TraesCS3D02G327600.1	3D	439,677,492–439,683,178	477	51.98	6.53
*TaDOG33*	TraesCS3D02G365200.3	3D	479,612,897–479,620,687	580	62.92	6.54
*TaDOG34*	TraesCS4A02G126300.2	4 A	162,423,186–162,427,249	335	37.35	7.87
*TaDOG35*	TraesCS4A02G183400.2	4 A	460,896,761–460,903,389	541	59.13	6.44
*TaDOG36*	TraesCS4B02G135000.1	4B	176,260,221–176,265,796	566	62.18	6.4
*TaDOG37*	TraesCS4B02G178600.1	4B	391,537,582–391,541,691	334	37.27	7.87
*TaDOG38*	TraesCS4D02G129900.1	4D	115,934,039–115,940,363	538	58.83	6.41
*TaDOG39*	TraesCS4D02G180200.4	4D	314,063,385–314,067,508	335	37.35	7.87
*TaDOG40*	TraesCS5A02G174200.1	5 A	367,556,709–367,565,775	467	51.25	7.02
*TaDOG41*	TraesCS5A02G265600.1	5 A	477,392,155–477,401,214	518	57.86	6.51
*TaDOG42*	TraesCS5B02G265300.1	5B	449,612,423–449,621,819	526	58.69	6.58
*TaDOG43*	TraesCS5D02G178800.1	5D	278,260,567–278,270,134	466	51.05	7.83
*TaDOG44*	TraesCS5D02G273500.1	5D	376,437,850–376,446,745	518	57.83	6.55
*TaDOG45*	TraesCS6A02G165800.1	6 A	166,982,263–166,991,094	466	50.95	7.01
*TaDOG46*	TraesCS6A02G248300.1	6 A	461,129,714–461,137,492	232	25.30	9.61
*TaDOG47*	TraesCS6B02G193200.2	6B	227,641,866–227,651,596	458	50.06	7.3
*TaDOG48*	TraesCS6B02G276400.1	6B	500,223,667–500,231,189	232	25.31	9.46
*TaDOG49*	TraesCS6D02G154400.1	6D	129,486,855–129,495,855	466	50.98	7.01
*TaDOG50*	TraesCS6D02G230400.1	6D	323,692,826–323,700,529	232	25.27	9.97
*TaDOG51*	TraesCS7A02G207100.1	7 A	169,397,681–169,401,164	443	48.31	7.86
*TaDOG52*	TraesCS7B02G114300.1	7B	132,168,398–132,171,701	448	48.46	7.36
*TaDOG53*	TraesCS7D02G209800.1	7D	167,584,688–167,588,228	448	48.44	7.36

### Phylogenetic analysis of the DOG genes

**Fig. 1:**
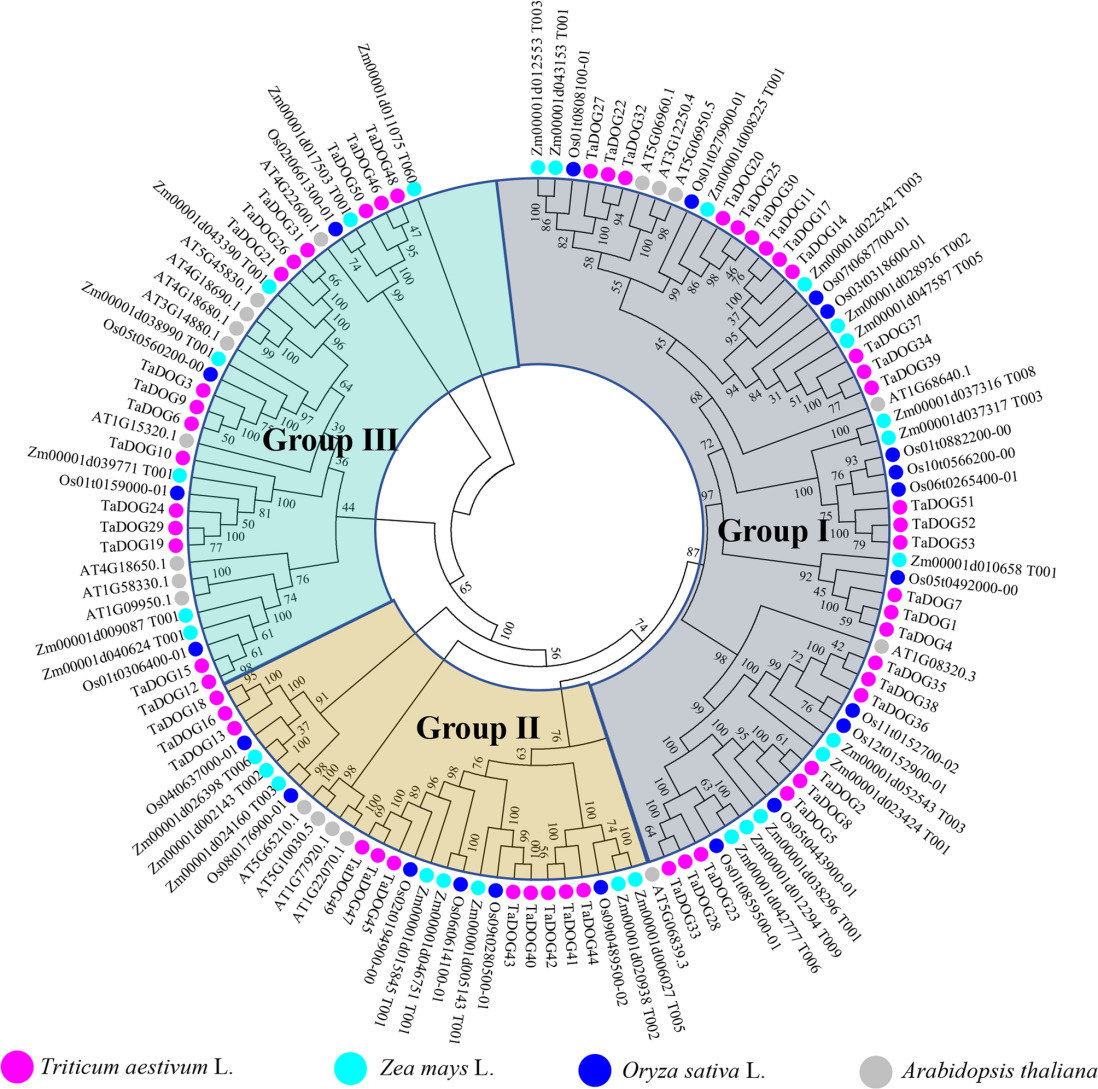
Phylogenetic analysis of the *DOG* gene family members. The purple solid circles represent DOG proteins in wheat (TaDOG); The indigo solid circles represent DOG proteins in maize; The blue solid circles represent DOG proteins in rice; The grey solid circles represent DOG proteins in Arabidopsis thaliana. The different colored sectors indicate different groups (or subgroups) of DOG proteins, including Group Ⅰ, Group Ⅱ and Group Ⅲ

The phylogenetic tree constructed with wheat, Arabidopsis, rice, and maize DOG proteins resolved into three major clades (Group Ⅰ - Group Ⅲ), supported by high bootstrap values (> 70%) (Fig. 1). Each group contains the *DOG* gene family members from four species. Among them, Group Ⅰ contained more DOG proteins. Protein sequence clustering indicates that *DOG* gene family members within the same group are highly similar, suggesting their similar functions and evolutionary processes. Compared with Arabidopsis, the wheat *DOG* gene family members were more closely related to those of rice and maize.

### Motif and gene structure analysis

**Fig. 2:**
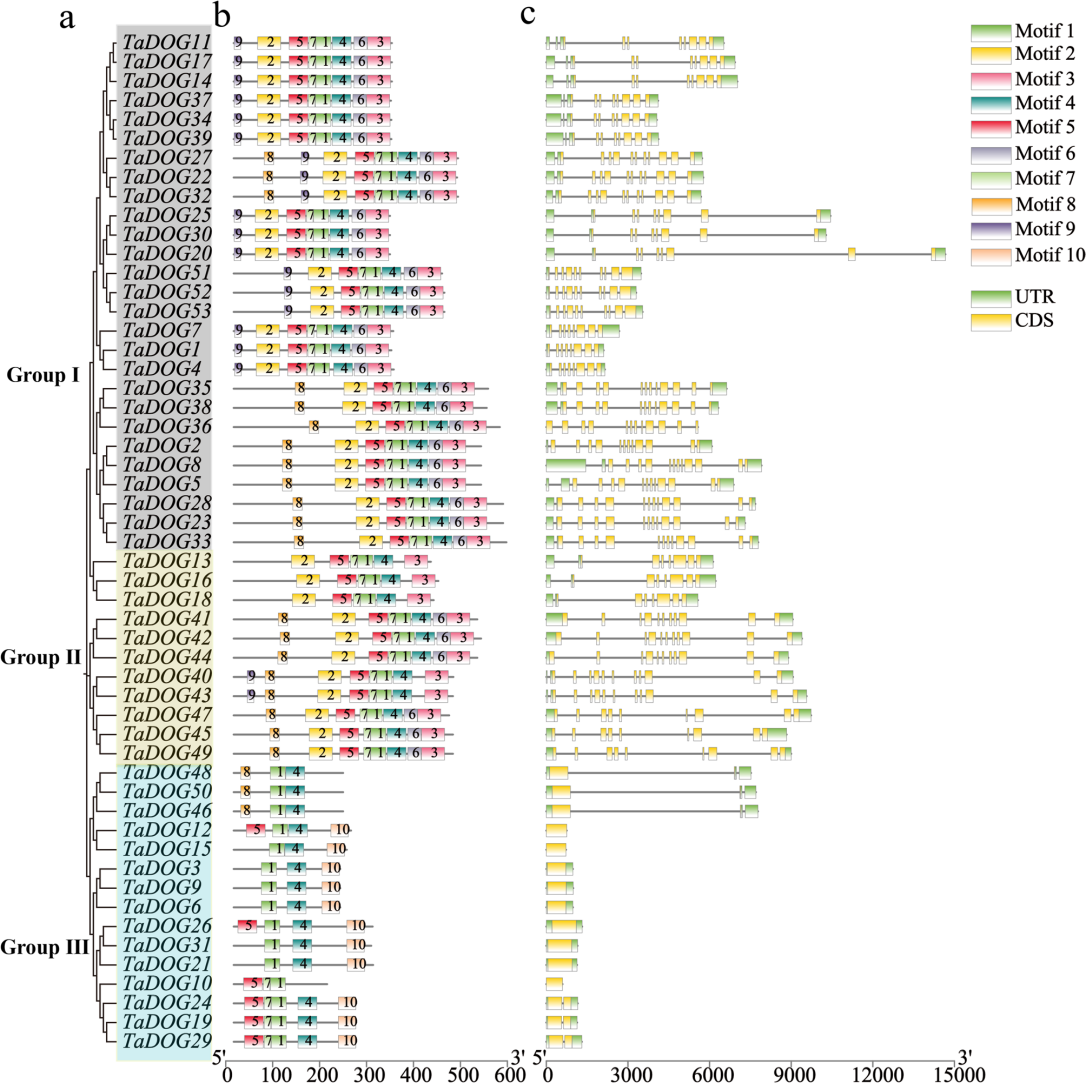
The analysis of the gene structure and protein sequence in wheat *DOG* gene family members. **a** The phylogenetic tree of TaDOG proteins, and clusters are indicated with different colors. **b** The Motif compositions of TaDOG proteins, and the 1–10 motifs are displayed in different colored boxes. **c** Exon-intron structures of the *TaDOG* genes, yellow boxes indicate untranslated regions; green boxes indicate exons, and black lines indicate introns

According to the clustering results of phylogenetic analysis (Fig. 2a), Motif and gene structure analysis were carried out. MEME analysis identified 10 conserved motifs (Motifs 1 ~ 10) across the DOG family (Fig. 2b). Motifs 1 and 4 were present in all members, corresponding to the core DOG1 domain, while other motifs showed group-specific distributions. For example, Motifs 1 ~ 7 exist simultaneously within Group Ⅰ, while Motifs 1 and 4 were enriched in Group Ⅲ. Gene structure analysis revealed that *TaDOG* genes had 1 ~ 12 exons, with Group Ⅰ members typically containing 8 ~ 12 exons, while *TaDOG* genes in Group Ⅲ had fewer (1 ~ 2 exons) (Fig. 2c). This structural variation may contribute to functional specialization.

### *Cis*-acting element analysis

**Fig. 3:**
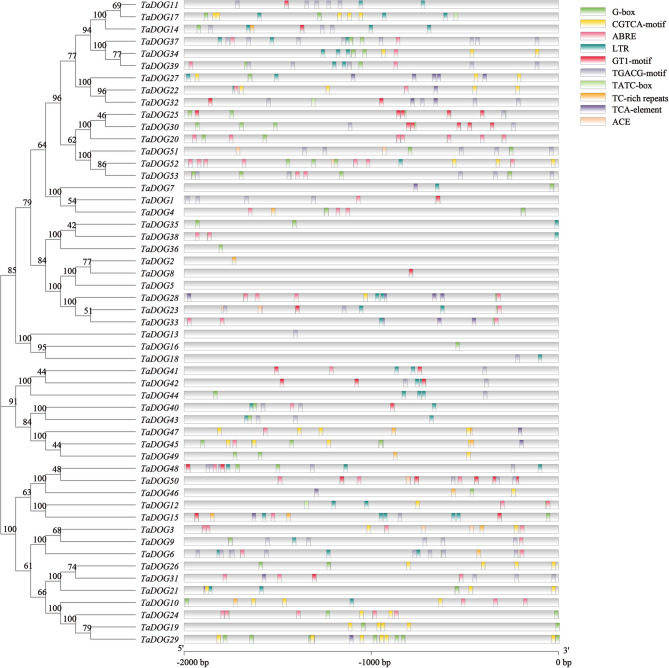
*Cis*-acting element analysis of the *DOG* gene family members in wheat. The abscissa represents the base sequence of 2000 bp upstream of the start codon “ATG”. Small rectangles in different colors represent different *Cis*-acting element, including the light responsiveness element (G-box, GT1-motif, ACE); the MeJA-responsiveness element (CGTCA-motif); the abscisic acid responsiveness element (ABRE); low-temperature responsiveness element (LTR); the MeJA-responsiveness element (TGACG-motif); the gibberellin-responsiveness element (TATC-box); the defense and stress responsiveness element (TC-rich repeats); the salicylic acid responsiveness element (TCA-element)

Promoter analysis identified numerous *cis*-acting elements related to hormone signaling and stress response (Fig. 3). ABA-responsive elements (ABREs) were found in 22.52% of *TaDOG* promoters, while MeJA-responsive elements (CGTCA-motif and TGACG-motif) were present in 36.56%. Besides, salicylic acid (TCA-element) and gibberellin-responsiveness (TATC-box) were present in 3.05% and 0.53%, respectively. Elements associated with low-temperature stress (LTR), light stress (G-box, GT1-motif, ACE), defense and stress responsiveness (TC-rich repeats) were also abundant. These developmental regulatory elements indicated potential roles in seed development.

### Genome collinearity analysis

**Fig. 4:**
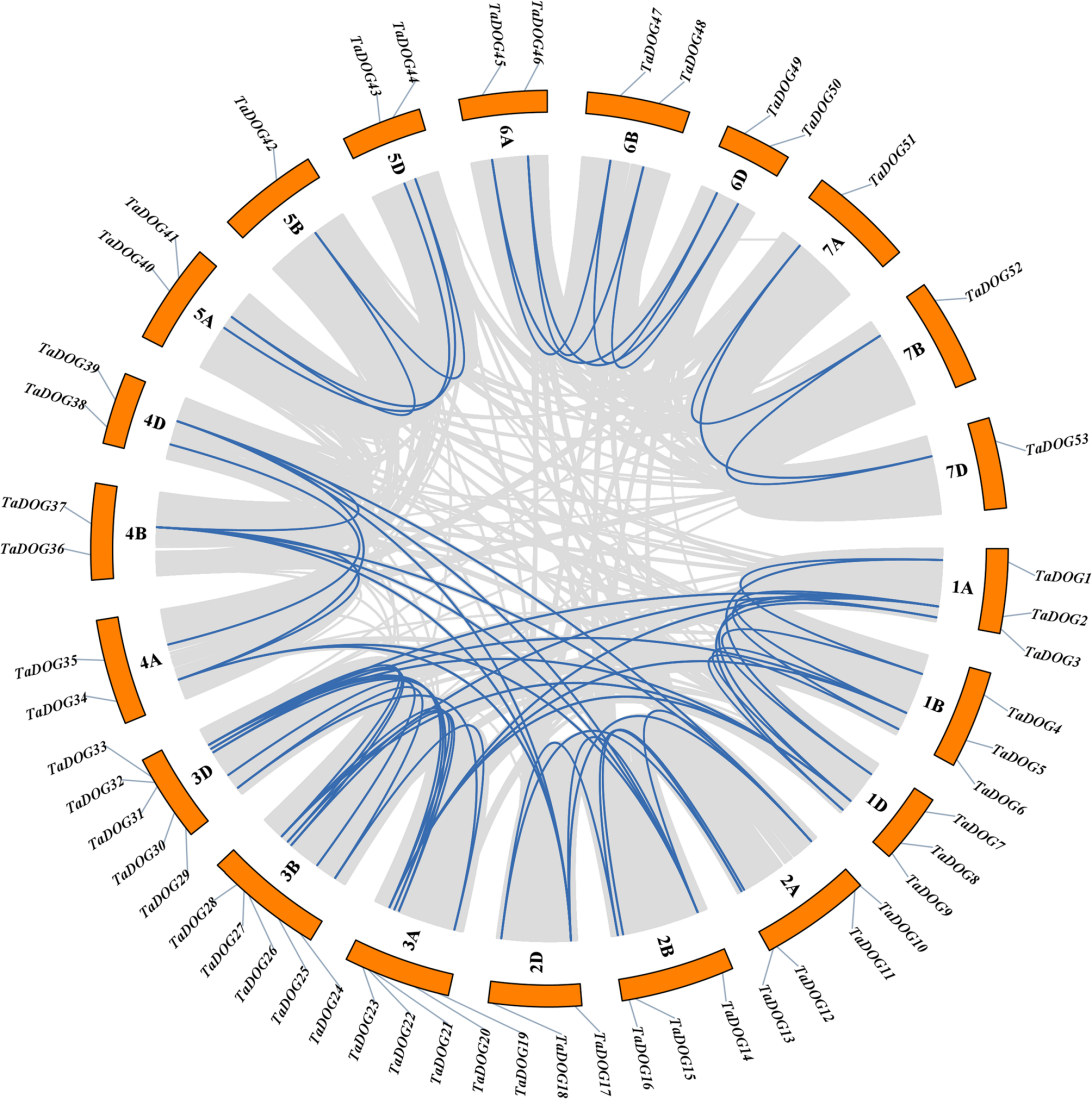
Chromosome localization and collinearity analysis of the *DOG* gene family members in wheat. The grey lines indicate all collinearity gene pairs in wheat, the highlighted blue lines indicate all *TaDOG* gene pairs

Intra-genomic collinearity analysis identified 124 segmental duplication events, and no tandem duplication events were found among wheat *DOG* genes (Fig. 4, Table S2). Almost all segmental duplication existed between homologous chromosomes and were involved in all 21 chromosomes of wheat. It suggested that whole-genome segmental duplication events played a major role in *DOG* gene expansion in wheat.

**Fig. 5:**
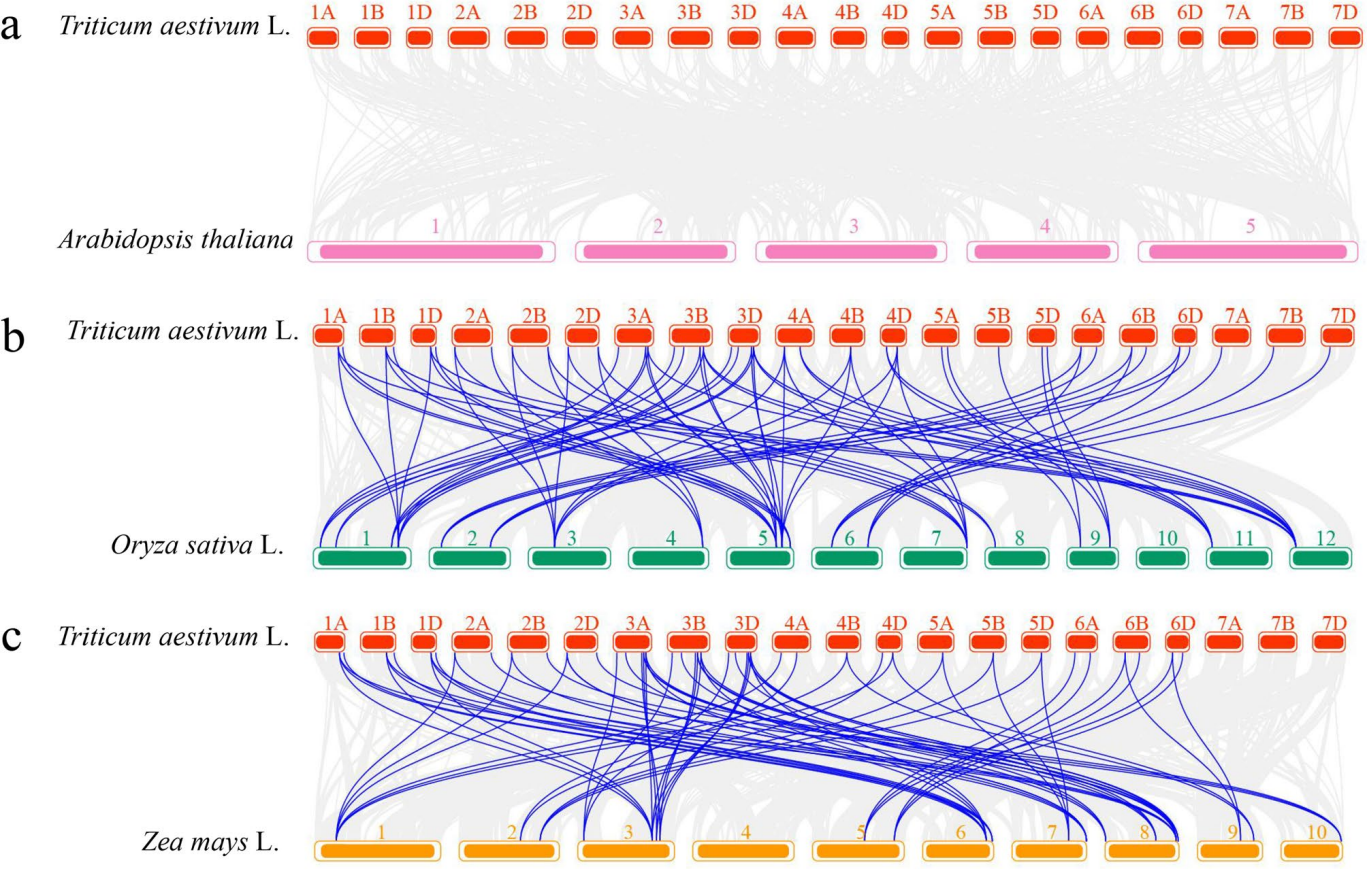
Evolutionary analysis of the wheat *DOG* gene family members between wheat and the other species. Gray lines in the background indicate the collinear blocks within wheat and other plant genomes, while the blue lines highlight the syntenic ARF gene pairs

Three comparative syntenic maps between the wheat genome and other three species (Arabidopsis, rice and maize) were conducted to understand the evolution mechanism of the *DOG* genes (Fig. 5). Synteny analysis with Arabidopsis, maize and rice revealed 0, 70, and 72 orthologous gene pairs, respectively. *TaDOGs* had a higher syntenic relationship with grass plants rice and maize. Especially, no syntenic relationship was found between wheat and Arabidopsis. 42 of the 53 *TaDOGs* had a syntenic relationship with 19 rice *DOG* genes (Table S3). Similarly, 46 of the 53 *TaDOGs* had a syntenic relationship with 20 maize *DOG* genes (Table S4). It is speculated that the *DOG* gene sequence fragments of grass plants such as wheat, rice and maize are highly conserved, and they have closer phylogenetic relationships. In addition, these genes have evolved from ancient *DOG* homologous genes.

### The expression patterns of TaDOGs in different tissues

**Fig. 6:**
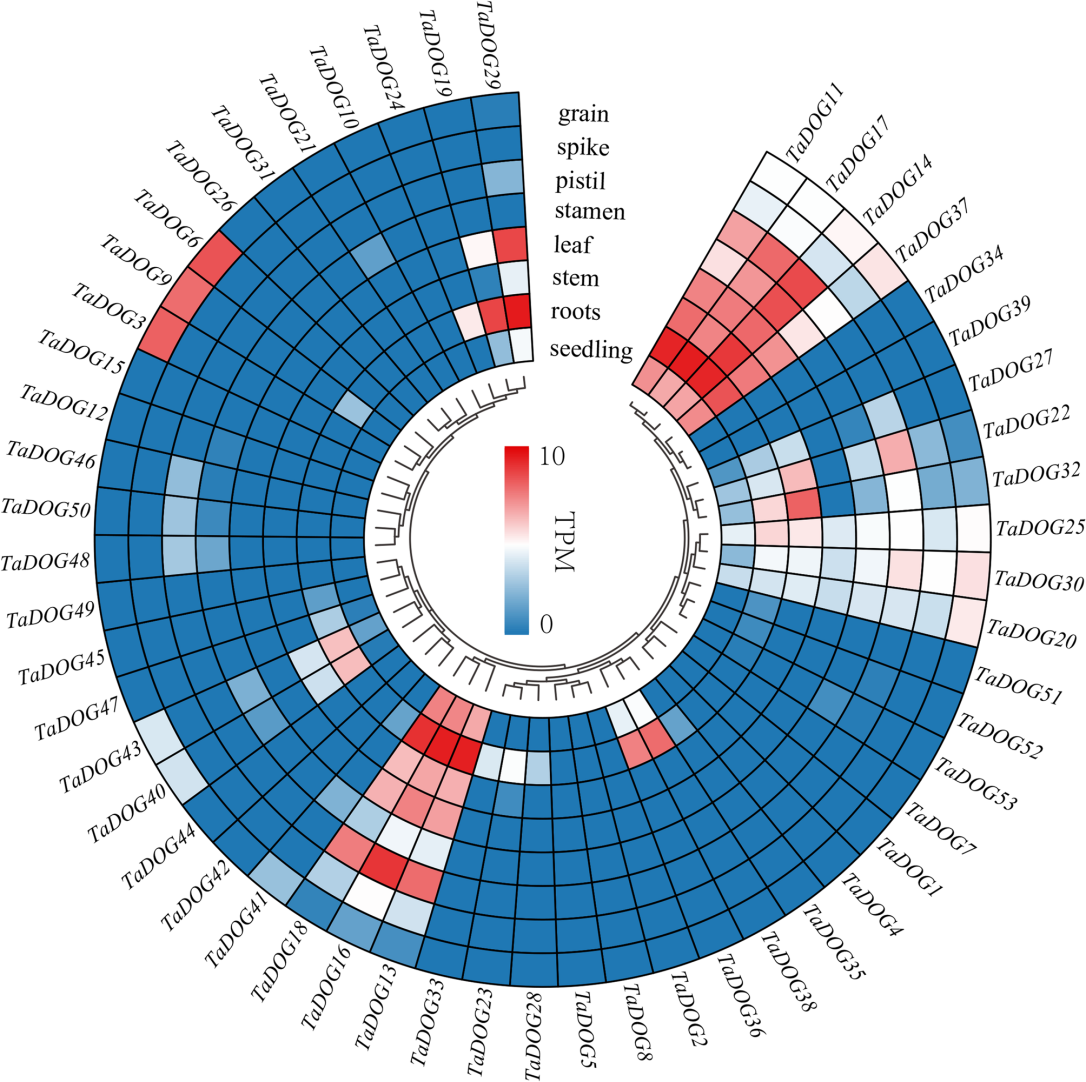
Expression profiles of *TaDOG* genes in various organs or tissues of wheat. Heatmap of expression profiles of *TaDOGs* in various organs or tissues of Chinese Spring from the Wheat Expression Browser (http://www.wheat-expression.com/). Gene expression levels were estimated by TPM values, and presented as log_2_-transformed normalized TPM

RNA-seq analysis revealed that *TaDOG* genes exhibited diverse expression profiles across different tissues (Fig. 6), with the data in Fig. 6 derived from 8 distinct wheat organs/tissues. Cluster analysis further classified these *TaDOG* genes into four typical expression clusters, whose characteristics and functional implications are described as follows: (1) Tissue-specific or developmentally regulated high expression: Some *TaDOGs* showed high expression exclusively in specific tissues or displayed dynamic expression changes during wheat development. For instance, *TaDOG3*, *TaDOG6*, and *TaDOG9* were highly expressed in grains, while *TaDOG19* exhibited high expression in roots. (2) Constitutive high expression across most tissues: A subset of *TaDOGs* maintained high expression levels in most tissues throughout wheat development, including *TaDOG11*, *TaDOG14*, *TaDOG17*, *TaDOG13*, *TaDOG16*, and *TaDOG18*. Given their broad and high expression patterns, these genes likely fulfill fundamental and essential roles in wheat developmental processes. (3) Ubiquitous but low expression: Another group of *TaDOGs* was expressed in all tissues during wheat development, albeit at relatively low levels (e.g., *TaDOG20*, *TaDOG25*, and *TaDOG30*). Despite their low expression abundance, these genes are also presumed to contribute to basic developmental processes in wheat. (4) Broadly low expression: A large number of *TaDOGs* showed extremely low expression in all examined tissues during wheat development, such as *TaDOG1*, *TaDOG4*, *TaDOG7*, and *TaDOG34*. Notably, the majority of *TaDOGs* fell into this category, and they are thought to play vital roles in the development of various wheat organs.

RNA-seq analysis showed that *TaDOG* genes exhibited diverse expression profiles across tissues (Fig. 6), which were derived from 8 different wheat organs/tissues. Cluster analysis grouped *TaDOG* genes into four typical expression clusters. (1) *TaDOGs* expressed highly only in specific tissues or their expression levels were changed during wheat development, such as *TaDOG3*, *TaDOG6* and *TaDOG9*, which were expressed highly in grain and *TaDOG1*9 which was expressed highly in roots. (2) *TaDOGs* which were expressed highly in most tissues during wheat development, such as *TaDOG11*, *TaDOG14*, *TaDOG17*, *TaDOG13*, *TaDOG16* and *TaDOG18*. These genes probably play basic important roles during wheat development. (3) *TaDOGs* which were expressed in all tissues during wheat development, but the expression levels were relatively lower, such as *TaDOG20*, *TaDOG25* and *TaDOG30*. They probably also play basic roles during wheat development. (4) *TaDOGs* expressed very lowly in all tissues during wheat development, such as *TaDOG1*, *TaDOG4*, *TaDOG7*, *TaDOG34* and so on. Most *TaDOGs* belong to this class and they play vital roles in various organ developments.

**Fig. 7:**
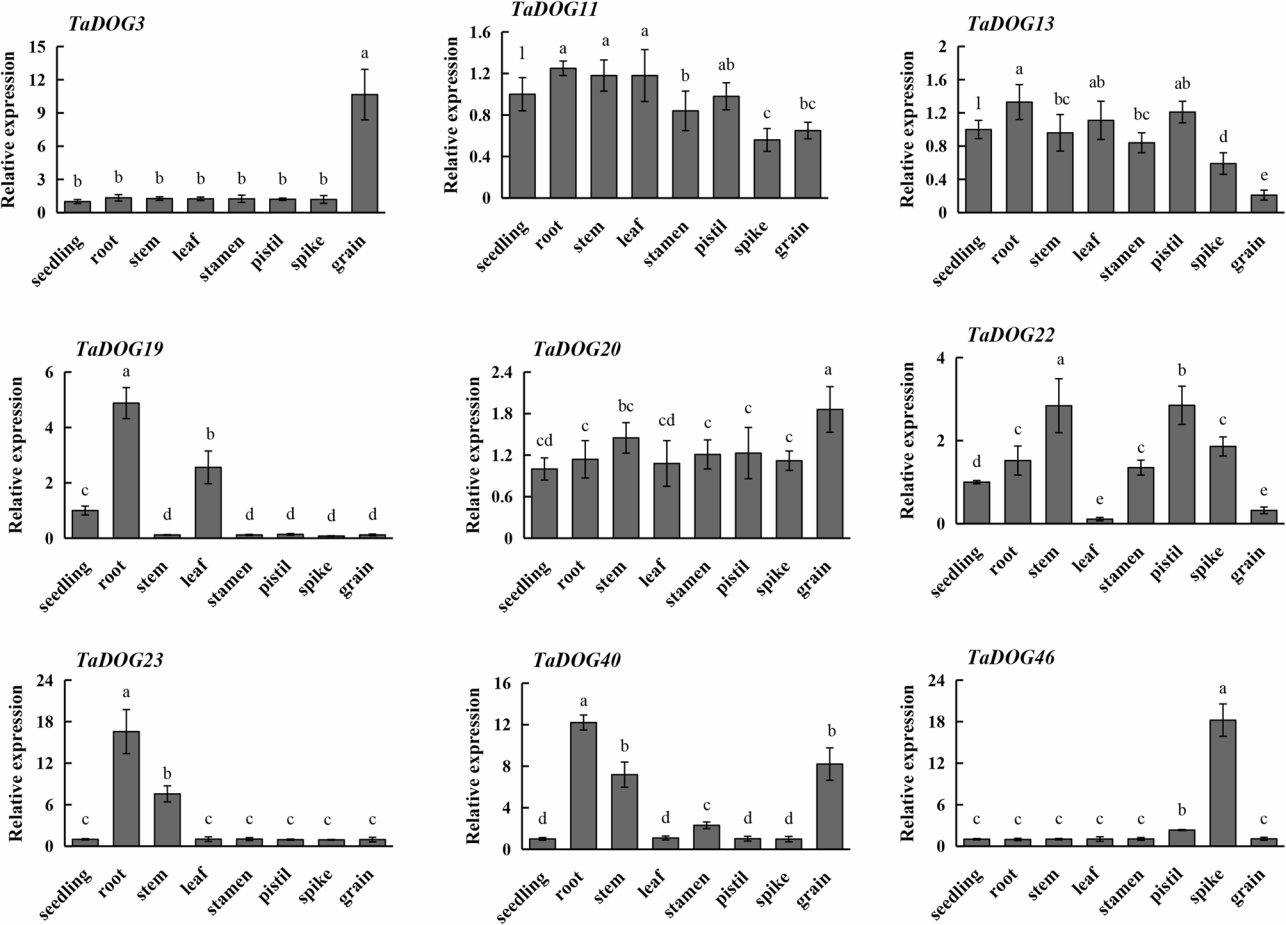
Gene expression analysis of wheat *DOG* gene family members in different tissues or organs. The seedling at three-leaf stage (the 45th day after wheat sowing), root, stem, leaf, stamen, pistil, spike, and grain at the anthesis stage (the 215th day after wheat sowing) were used to carry out RT-PCR

Since the homoeoalleles of most tri-genes exhibited similar expression levels in wheat, universal primers were used to analyze the expression of *TaDOG* homoeoallele genes [30]. Here, a total of nine representative *DOG* genes (including *TaDOG3*, *TaDOG11*, *TaDOG13*, *TaDOG19*, *TaDOG20*, *TaDOG22*, *TaDOG23*, *TaDOG40* and *TaDOG46*) were verified by RT-PCR for gene expression levels in different wheat tissues or organs (Fig. 7). The results indicated that the expression trends of these genes in different tissues or parts of wheat were consistent with the data in RNA-seq (Fig. 6). More importantly, *TaDOG3/6/9* shows obvious tissue specificity and has a significantly high expression level in wheat grain development. More importantly, *TaDOG3/6/9* (including its homoeologous genes) shows obvious tissue specificity and has a significantly high expression level in wheat grain development. It is speculated that it can be used as a landmark gene to test the dormancy degree of seeds.

### Hormone-responsive expression under MeJA and ABA treatments

The *cis*-acting element analysis showed that a number of hormone response-related *cis*-elements existed in the promoter regions of *TaDOG* genes, especially those related to MeJA and ABA responsiveness (Table S5).

**Fig. 8:**
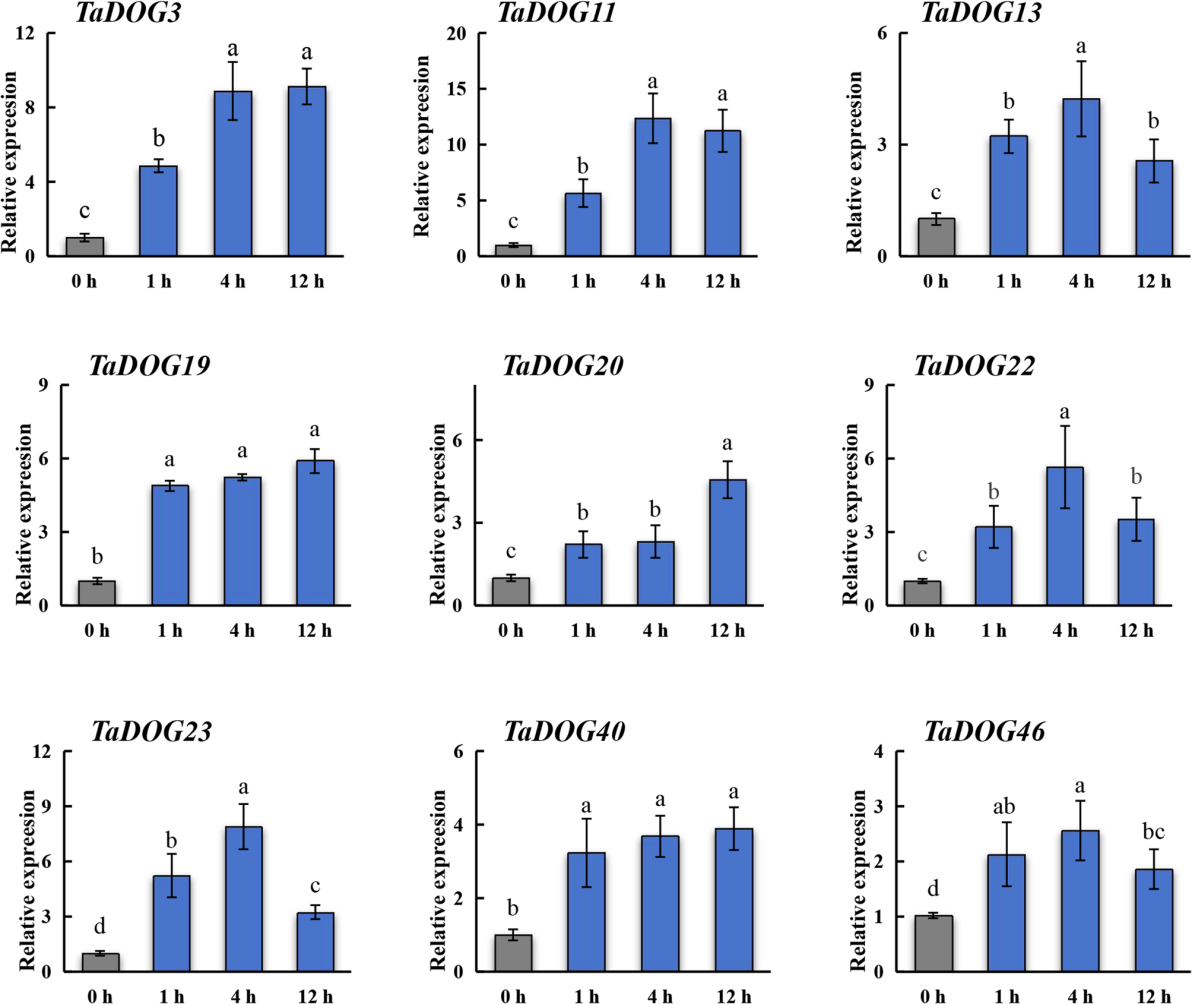
Gene expression analysis of wheat *DOG* gene family members under MeJA stress. Data were normalized to *actin* gene and vertical bars indicated standard deviation. Statistical significance was evaluated using Student’s *t*-tests, different letters indicate a significant difference between samples (*P* < 0.05)

**Fig. 9:**
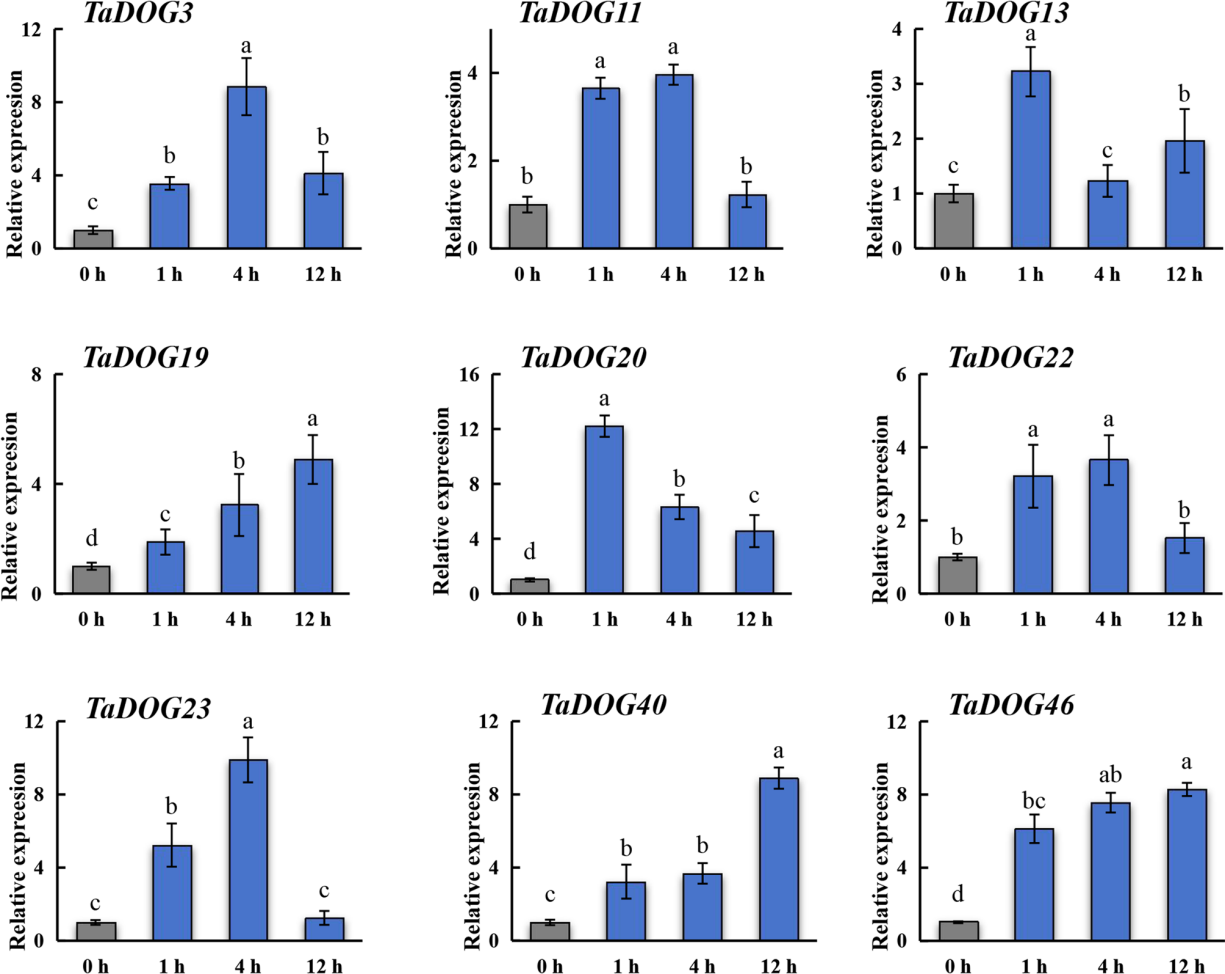
Gene expression analysis of wheat *DOG* gene family members under ABA stress. Data were normalized to *actin* gene and vertical bars indicated standard deviation. Statistical significance was evaluated using Student’s *t*-tests, different letters indicate a significant difference between samples (*P* < 0.05)

RT-PCR analysis revealed a clear dynamic expression changeof *TaDOG* genes in response to MeJA (Fig. 8). Following MeJA treatment, 9 genes were upregulated (> 2 fold) at 1 h. Among them, *TaDOG4*, *TaDOG11* and *TaDOG23* expression changed more than 5 fold at 1 h. Here, the expression trend of the *DOG* gene in response to MeJA treatment can be roughly divided into two categories. One is that the expression level of the *DOG* gene shows a trend of first increasing and then decreasing within 12 h, including *TaDOG11*/*1*3/*22*/*23*/46. The other is that the expression level of the *DOG* gene continues to rise within 12 h with the change of time, such as *TaDOG3*/*19*/*20*/*40*. The dynamic expression profiles of the *DOG* genes under ABA treatment showed that different family members showed different temporal expression patterns after ABA treatment (Fig. 9). It was significantly upregulated at specific time points (1 h, 4 h and 12 h), showing a distinct early response pattern. For instance, *TaDOG13* rose sharply (peak) at 1 h, then dropped at 4 h, and partially recovered at 12 h. *TaDOG11* remained highly expressed at both 1 h and 4 h, while *TaDOG3* and *TaDOG22* reached their peaks at 4 h. *TaDOG20* rose sharply (peak) at 1 h and then declined. *TaDOG19* and *TaDOG46* exhibit a delayed response pattern. Over time, the gene expression level gradually upregulates, reaching a peak or persisting at 12 h. Similarly, *TaDOG40* shows delayed induction and significant increases at 12 h. *TaDOG23* reached its peak at 4 h but dropped sharply to near the baseline level at 12 h, indicating a brief response to ABA induction.

These different expression dynamics indicate the existence of functional specialization within the *TaDOG* family. This difference in time regulation indicates that different *TaDOG* genes may mediate ABA signaling at different stages and may coordinate the delay of germination through complementary or sequential regulatory effects.

### Negative correlation between TaDOG3 expression and PHS rate

**Fig. 10:**
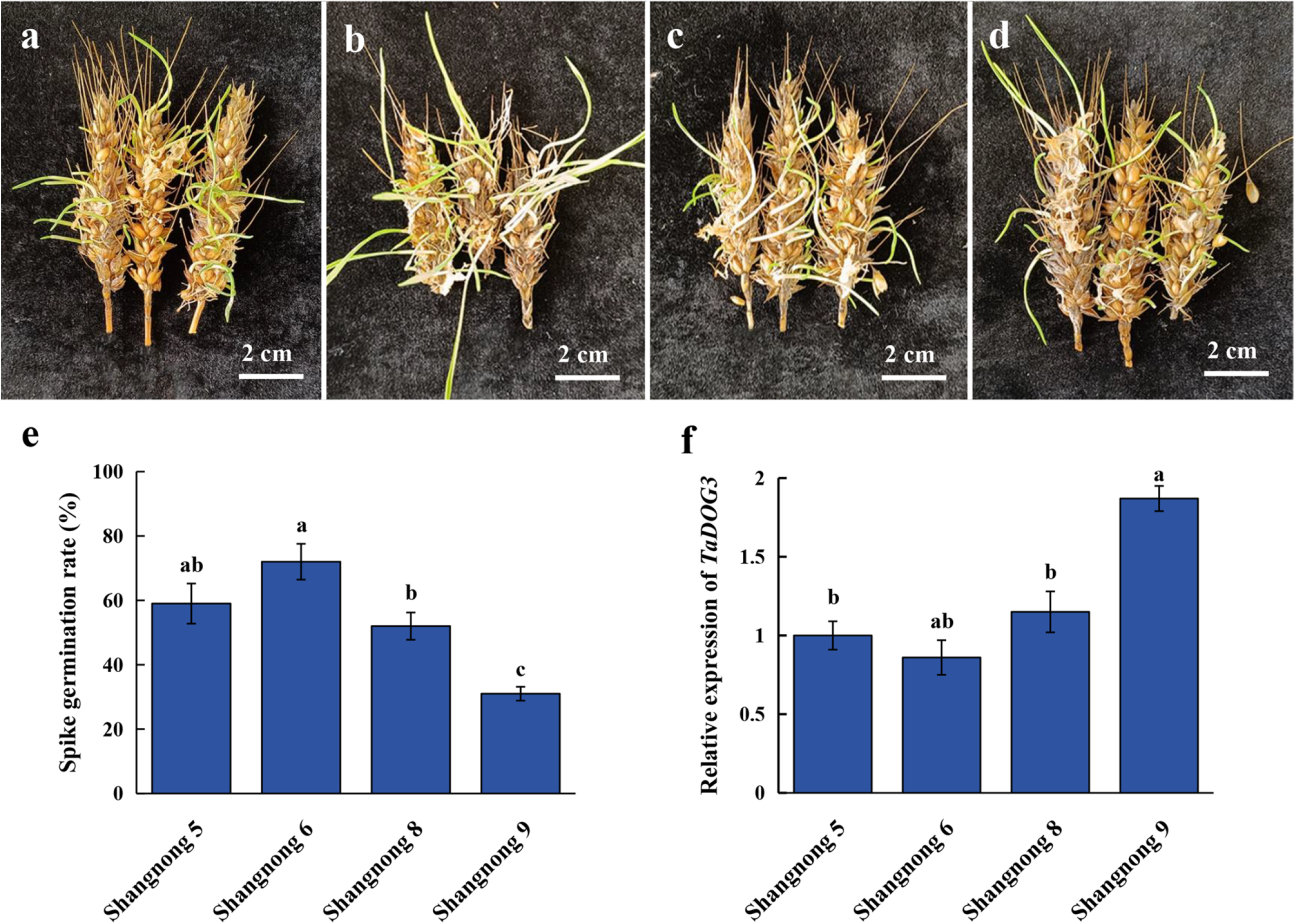
Investigation of PHS in different wheat cultivars. **a** Phenotypic diagram of Shangnong 5 in the PHS trial. **b** Phenotypic diagram of Shangnong 6 in the PHS trial. **c** Phenotypic diagram of Shangnong 8 in the PHS trial. **d** Phenotypic diagram of Shangnong 9 in the PHS trial. **e** PHS rate in different wheat cultivars. **f** relative expression of *TaDOG3* in different wheat cultivars. Statistical significance was evaluated using Student’s *t*-tests, different letters indicate a significant difference between samples (*P* < 0.05)

Four wheat varieties with highly similar genetic backgrounds were selected for the spike germination test, and there were significant differences in spike germination phenotypes of wheat (Fig. 10a-e). The PHS rate in Shangnong 6 was long and dense, and the germination degree of the whole spike was the highest (72.12%). In Shangnong 9, the germination of spike grain was short and few, and the PHS rate was the lowest (31.03%). Shangnong 5 and Shangnong 8 showed an intermediate PHS rate of 59.18% and 53.19%, respectively. These phenotypic differences directly reflect the differences in PHS rate among varieties. The relative expression levels of *TaDOG3* were detected respectively in different wheat varieties (Fig. 10f). The results showed that the relative expression level of *TaDOG3* was the highest in Shangnong 9 and the lowest in Shangnong 6. The expression trend is completely opposite to the germination rate. It indicates that the expression level of the *TaDOG3* gene is negatively correlated with the PHS rate.

## Discussion

### Evolutionary expansion and structural diversification of TaDOG genes

In this study, members of the *DOG* gene family in wheat were systematically identified. Meanwhile, members of the *DOG* gene families of *Arabidopsis thaliana*, rice and corn were also identified (Fig. 1). The identification of 53 *TaDOG* genes in hexaploid wheat reflects a significant expansion of the *DOG1* family compared to diploid species (e.g., 19 *AtDOG* genes in *Arabidopsis*). This expansion is primarily driven by whole-genome segmental duplications (124 events) (Fig. 4), consistent with wheat’s evolutionary history of two rounds of allopolyploidization [19]. Structural analysis revealed marked differences in exon-intron organization [31]. Group Ⅰ genes (8 ~ 12 exons) in phylogenetic trees (Fig. 1) likely encode multifunctional proteins with complex regulatory interactions, while Group Ⅲ genes (1 ~ 2 exons) may act as fast-responsive regulators [32]. The structural complexity can create extensive opportunities for alternative splicing, a mechanism well-documented to generate proteomic diversity by producing multiple transcript variants from a single gene locus [33]. Similar to the *Arabidopsis DOG1* gene, which produces five transcript variants through alternative splicing [8]. The complex intronic regions of Group Ⅰ genes may further enhance regulatory flexibility by providing additional binding sites for transcription factors and cis-regulatory elements, facilitating fine-tuning of gene expression in response to fluctuating environmental conditions [34]. In striking contrast, the simplified structure of Group Ⅲ *TaDOG* genes (1 ~ 2 exons) suggests a specialized role as rapid-response regulators. Genes with fewer introns are generally associated with faster transcription and translation rates due to minimized pre-mRNA processing requirements [34]. This structural efficiency is particularly advantageous for genes involved in immediate stress responses, where rapid protein synthesis is critical for adaptive fitness. Such diversification highlights how the *DOG* gene family has adapted to meet the dual demands of precise regulatory control and environmental responsiveness essential for wheat seed survival and propagation [35]. Conserved motifs (e.g., Motif 1 and 4, core *DOG1* domains) across all members confirm functional conservation, whereas group-specific motifs (e.g., Motifs 1 ~ 7 in Group Ⅰ) suggest specialized roles. It is speculated that they had played a fundamentally important role in *TaDOG* evolution [36].

The uneven chromosomal distribution (e.g., 15 genes on chromosome 3) and phylogenetic clustering into three groups imply subfunctionalization during evolution. The closer syntenic relationship with rice and maize (72 and 70 orthologous pairs, respectively) than with *Arabidopsis* (0 pair) aligns with monocot-dicot divergence, supporting the conserved evolutionary trajectory of the *DOG* genes in grass species [10]. Promoter *cis*-elements enriched in ABA, MeJA, and stress-responsive motifs (e.g., ABRE, CGTCA-motif) provide a mechanistic link between *TaDOG* regulation and environmental signals [37]. The high frequency of MeJA-responsive elements (36.56%) and ABA-responsive elements (22.52%) correlates with the dynamic expression shifts observed under these hormones, indicating that *TaDOG* genes integrate multiple signaling pathways [38, 39]. These results indicate that the *TaDOG* gene family actively responds to exogenous hormone treatment. It may change the balance among hormones by regulating the seed’s sensitivity to ABA or jasmonic acid, thereby participating in the regulation of seed dormancy and germination [40, 41].

### Expression patterns and biological functions of TaDOG genes

The RNA-seq-based dissection of *TaDOG* expression across eight wheat tissues, validated by RT-PCR, reveals a complex functional diversification. In plants, gene expression patterns are tightly linked to biological roles: ubiquitously expressed genes often support fundamental processes, while tissue-specific genes mediate specialized functions [42]. For *TaDOG* genes, tissue-specific expression profiles suggest that *TaDOG* genes have diversified roles in wheat development. Seed-preferential genes (e.g., *TaDOG3/6/9*) may function in seed dormancy and germination (Figs. 6 and 7), analogous to Arabidopsis *DOG1*, while vegetative-preferential genes could be involved in growth regulation or stress adaptation [43]. *AtDOG1* is predominantly expressed in developing seeds, where it delays germination by integrating ABA signaling and epigenetic regulation [5]. The high expression of *TaDOG3* during wheat grain development suggests a conserved function in maintaining seed dormancy, potentially mitigating pre-harvest sprouting [44]. Besides, root-specific *TaDOG19* may mediate ABA-dependent stress responses in roots, as ABA signaling orchestrates root architecture and drought tolerance [45]. *TaDOG11/14/17* are highly expressed across most tissues, implying housekeeping or core regulatory roles. In Arabidopsis, some *DOG1*-like genes are constitutively expressed and interact with multiple signaling pathways [46]. For wheat, these genes likely stabilize basic developmental processes (e.g., cell cycle regulation, hormone homeostasis), which are essential across tissues. Their conserved expression across wheat’s three subgenomes (via homoeoallele expression consistency) further supports their indispensability [30]. Some *DOG* genes, such as *TaDOG20/25/30*, are broadly expressed but at low levels, suggesting they fine-tune development rather than drive core processes. In polyploid genomes, these genes often arise from subfunctionalization, where duplicated genes partition ancestral functions [47]. For *TaDOGs*, low-level ubiquitous expression may allow dependent responses to environmental cues (e.g., temperature, moisture), aligning with *cis*-elements related to stress and hormone signaling [48]. Clusted genes (*TaDOG1/4/7* and so on) show negligible expression across tissues, a common feature in polyploid gene families [49]. These may represent pseudogenes, or their expression could be induced by specific stimuli not captured in our analysis. Alternatively, they might provide genetic redundancy, a hallmark of polyploidy-driven buffering against mutations [50].

It is worth noting that the grain-specific expression of *TaDOG3* (and its homoeoalleles) makes it a promising biomarker for seed dormancy. In Arabidopsis thaliana, the expression level of *AtDOG1* is related to the depth of dormancy, and high expression delays germination [51]. Therefore, the expression of the *TaDOG*3 gene in wheat can be quantified to predict the risk of pre-harvest sprouting in wheat [52].

### Hormonal response change and functional specialization of the TaDOG gene

Plant hormone signaling is orchestrated by a complex network of *cis*-acting elements and transcription factors [48]. Hormone-responsive expression under MeJA and ABA indicates that *TaDOG* genes are integrated into complex regulatory networks, potentially mediating cross-talk between stress and developmental pathways [53]. The enrichment of ABA-responsive elements (ABREs) and MeJA-responsive motifs (CGTCA/TGACG) in *TaDOG* promoters provides a mechanistic link between hormone perception and transcriptional activation.

The expression of *TaDOG* induced by MeJA is classified into “transient induced decrease” (such as *TaDOG11*, *TaDOG*23) and “sustained upregulation” (such as *TaDOG*3, *TaDOG1*9), which reflects the functional specialization of the *TaDOG* gene. Transient induction genes may mediate early signal propagation: Their rapid peaks are consistent with the role of MeJA in triggering immediate defense responses [54]. On the contrary, continuous expression implies the role of long-term physiological regulation, such as enhancing seed dormancy under abiotic stress to prevent germination. This differentiation may stem from the complexity of the active elements in the promoter region [55]. The diversity of this regulatory expression pattern reflects the different adaptabilities of *TaDOGs* during their evolution. ABA-induced *TaDOG* expression patterns reveal a temporal hierarchy in dormancy regulation, including early (1–4 h), transient (peak at 4 h) and delayed (12 h). Early responders (e.g., *TaDOG1*3, *TaDOG11*) likely initiate dormancy: in Arabidopsis, At*DOG1* enhances ABA sensitivity to delay germination [56], and their rapid induction suggests a role in “locking” seeds into dormancy. Transient responders (e.g., *TaDOG*23) act as “switches”, preventing over-activation of ABA signaling and potential developmental arrest. Delayed responders (e.g., *TaDOG1*9, *TaDOG*46) may sustain dormancy via epigenetic mechanisms [57]. This temporal gradient mirrors the sequential phases of seed dormancy (induction, maintenance, and release) with distinct *TaDOGs* governing each stage. Such precision is critical for wheat, as misregulated dormancy leads to PHS, a major yield constraint [58].

### TaDOG3 can be used as a marker gene to detect the degree of spike germination

PHS is a key adverse condition in wheat production that leads to yield loss and deterioration of processed products [59]. Against the background of global warming, the frequency of precipitation during wheat harvest has increased in recent years, and the risk of PHS has been further aggravated. It is important to analyze the genetic mechanism of PHS for breeding resistance improvement [53]. This study selected four wheat varieties (Hybrid combinations have the same paternal or maternal material) with highly similar genetic backgrounds (Table S6), effectively eliminating the interference of complex genetic interactions and more clearly revealing the association between *TaDOG3* and PHS.

In *Arabidopsis thaliana*, *DOG1* is the core regulatory gene of seed dormancy. It enhances dormancy by delaying germination, and its expression level is positively correlated with the depth of dormancy [5, 60]. As a *DOG1* homologous gene, wheat *TaDOG3* shows a pattern of “high expression in low-PHS varieties and low expression in high-PHS varieties” among the four varieties, suggesting that it conservatively inherits the functions of “promoting dormancy and inhibiting germination”. In similar studies, the wheat *TaDOG1*-like gene has also been shown to be significantly associated with dormancy. It further supports the hypothesis that *TaDOG3* is involved in the regulation of PHS [14]. Since the genetic background of the tested varieties is highly similar, the phenotypic differences are more likely to be due to the differentiation of *TaDOG3* expression regulation (such as *cis*-element variations in the promoter region and differences in transcription factor binding). Rather than complex genetic interactions, this lays the foundation for subsequent gene localization and functional verification. The core of seed dormancy and germination is the dynamic balance among hormones: ABA promotes dormancy and GA promotes germination [61]. Wheat variety Shangnong 9 with high expression of *TaDOG3* has strong dormancy and a low PHS rate. It is speculated that it may enhance ABA signals (such as promoting ABA synthesis and inhibiting ABA degradation) or suppress GA signals (such as down-regulating GA synthesis genes), and delay grain germination [62]. The latest research has found that the interaction between wheat *TaPP2C-a6* and *TaDOG1* affects seed dormancy by regulating the ABA signaling pathway [63]. *TaDOG3* might synergistically act with TaPP2C-a6 to form an ABA signal regulation module, further enhancing dormancy. Furthermore, *DOG1* in *Arabidopsis* thaliana interacts with the phosphatase AHG1/AHG3 to inhibit the negative regulatory factor of ABA signaling, suggesting that *TaDOG3* may enhance ABA sensitivity and inhibit germination through a similar mechanism [64].

In this study, *TaDOG3* demonstrated a stable “expression-phenotype” association among varieties with similar genetic backgrounds, providing a direct target for molecular marker-assisted breeding. However, it should be noted that the function of *TaDOG3* may be regulated by environmental factors [38]. Therefore, genotype-environmental interaction studies need to be carried out to clarify the gene effects in different ecological regions and avoid the environmental dependence of molecular markers.

## Conclusion

This study identifies 53 *TaDOG* genes in wheat, revealing their evolutionary expansion via segmental duplication and functional diversification through structural and expression divergence. Promoter *cis*-elements and hormone-responsive link *TaDOGs* to ABA/MeJA signaling, while tissue-specific expression, especially grain-enriched *TaDOG*3—suggests roles in seed dormancy regulation. These findings lay a foundation for functional validation of *TaDOGs* in wheat dormancy and stress adaptation, offering potential genetic targets for breeding programs aiming to optimize seed germination traits.

## Supplementary Information

Supplementary Material 1: Table S1: List of primers used for *TaDOG* genes expression analysis; Table S2: One-to-one orthologous relationships between wheat and wheat; Table S3: One-to-one orthologous relationships between wheat and rice; Table S4: One-to-one orthologous relationships between wheat and maize; Table S5: The number of *cis*-acting elements in the promoter region of *TaDOG* genes; Table S6: The main information of four wheat varieties in this study.

## Data Availability

All data in this study can be found in public databases and Supplementary Materials. The name and approval numbers of four Chinese winter wheat varieties by China government in this study are Shangong 5 (unreleased), Shangnong 6 (released no. Yushenmai 20220077), Shangnong 8 (released no. Yushenmai 20220080), and Shangnong 9 (released no. Guoshenmai 20243004). These wheat varieties can be obtained from the Shangqiu Academy of Agricultural and Forestry Sciences. Supplemental information is provided within this manuscript or supplementary information files.

## Abbreviations

ABA: Abscisic acid
BR: Brassinosteroids
CK: Cytokinins
*DOG*: *Delay of germination 1*
ET: Ethylene
GA: Gibberellins
HMM: Hidden Markov model
IAA: Auxin
MeJA: Methyl jasmonate
NJ: Neighbor-joining
PHS: Pre-harvest sprouting
SA: Salicylic acid
SL: Strigolactones
TPM: Transcripts per million
